# An empirical analysis of dealing with patients who are lost to follow-up when developing prognostic models using a cohort design

**DOI:** 10.1186/s12911-021-01408-x

**Published:** 2021-02-06

**Authors:** Jenna M. Reps, Peter Rijnbeek, Alana Cuthbert, Patrick B. Ryan, Nicole Pratt, Martijn Schuemie

**Affiliations:** 1 Janssen Research and Development, Titusville, NJ USA; 2 Department of Medical Informatics, Erasmus University Medical Center, Rotterdam, The Netherlands; 3 South Australian Health and Medical Research Institute (SAHMRI), Adelaide, SA Australia; 4 Quality Use of Medicines and Pharmacy Research Centre, Sansom Institute, School of Pharmacy and Medical Sciences, University of South Australia, Adelaide, SA Australia

**Keywords:** Prognostic model, Loss to follow-up, Censoring, PatientLevelPrediction, Best practices, Model development

## Abstract

**Background:**

Researchers developing prediction models are faced with numerous design choices that may impact model performance. One key decision is how to include patients who are lost to follow-up. In this paper we perform a large-scale empirical evaluation investigating the impact of this decision. In addition, we aim to provide guidelines for how to deal with loss to follow-up.

**Methods:**

We generate a partially synthetic dataset with complete follow-up and simulate loss to follow-up based either on random selection or on selection based on comorbidity. In addition to our synthetic data study we investigate 21 real-world data prediction problems. We compare four simple strategies for developing models when using a cohort design that encounters loss to follow-up. Three strategies employ a binary classifier with data that: (1) include all patients (including those lost to follow-up), (2) exclude all patients lost to follow-up or (3) only exclude patients lost to follow-up who do not have the outcome before being lost to follow-up. The fourth strategy uses a survival model with data that include all patients. We empirically evaluate the discrimination and calibration performance.

**Results:**

The partially synthetic data study results show that excluding patients who are lost to follow-up can introduce bias when loss to follow-up is common and does not occur at random. However, when loss to follow-up was completely at random, the choice of addressing it had negligible impact on model discrimination performance. Our empirical real-world data results showed that the four design choices investigated to deal with loss to follow-up resulted in comparable performance when the time-at-risk was 1-year but demonstrated differential bias when we looked into 3-year time-at-risk. Removing patients who are lost to follow-up before experiencing the outcome but keeping patients who are lost to follow-up after the outcome can bias a model and should be avoided.

**Conclusion:**

Based on this study we therefore recommend (1) developing models using data that includes patients that are lost to follow-up and (2) evaluate the discrimination and calibration of models twice: on a test set including patients lost to follow-up and a test set excluding patients lost to follow-up.

## Background

Prediction models in healthcare can be used to identify patients who have a high risk of developing some undesirable outcome. An outcome is the occurrence of some medical event of interest and when implementing binary classification, patients are either labelled as having the outcome during some time-at-risk period or not having the outcome during the time-at-risk. Examples include the development of a new illness or illness progression, experiencing some adverse event and achieving some treatment response or adherence. Patients that are deemed as being at high-risk of an outcome can then be targeted for suitable interventions with the aim of reducing their risks. For example, numerous risk models are being clinically used to identify patients with a high risk of cardiovascular issues who may benefit from modification of blood lipids [1]. Prediction models address the patient’s question: ‘what is my probability of developing < insert outcome > during the next N years?’. However, many developed prediction models removed patients from the training data who left the database before the N year follow-up and therefore implicitly answered ‘what is my probability of developing < insert outcome > during the next N years given I remain in the data’. Loss to follow-up is the situation where a patient enters into a cohort study but stops being observable before the end of the study (e.g., they are not observed during the full time-at-risk period). Sometimes the cause of leaving the study is unknown. Many published papers did not investigate the impact that loss to follow-up may have on their model [2] and this has been highlighted as a challenge is risk prediction development [2].

Our recent framework for standardizing the development of patient-level prediction models [3] recommends defining some index date for each patient where the data prior to index are used to construct potential predictors and the data post index are used to identify whether the patient has the health outcome of interest during some follow-up period. The prediction question can be standardized into three parts: (1) the target population (the patients you want to apply the model to) and an index date when they enter the cohort, (2) the outcome (the medical event you want to predict) and (3) the time-at-risk (a time period relative to the target cohort index date where you wish to predict the outcome occurring). The prediction problem becomes: ‘Predict which patients in < Target Cohort > will experience < outcome > during the < time-at-risk > following target cohort entry.’ For example, we may wish to ‘predict which patients with depression who are pharmaceutically treated will experience nausea 1 day until 3 years after they are first diagnosed with depression’.

Sometimes patients are not observed for the complete time-at-risk period due to numerous reasons. Possible reasons include that they may change insurance, relocate to outside the database capture area, or die during the time-at-risk period. Continuing with the example, some patients with depression may change insurance, they may move to another country or they may die from other illnesses within the 3 years. We refer to these patients as being ‘lost to follow-up’ as they were not observed for the complete time-at-risk. There are four possibilities for each patient in training data: (1) having complete follow-up and no record of the outcome during time-at-risk means the patients is a ‘non-outcome’ patient, (2) having complete follow-up and a record of the outcome during time-at-risk means the patients is an ‘outcome’ patient, (3) having incomplete follow-up and a record of the outcome during the partially observed time-at-risk means the patients is an ‘outcome’ patient or (4) having incomplete follow-up and no record of the outcome during the partially observed time-at-risk means the patient’s label is unknown as they could have the outcome after being lost to follow-up. Should the patients who are lost to follow-up be included in training data, potentially making the labels noisy, or should they be excluded, which might cause generalizability issues or impact the model due to the data containing less patients with the outcome?

Researchers developing prediction models are faced with various design choices which may have significant impacts on the model performance. Some guidelines have been proposed for certain best practices in developing patient-level prediction models such as best practices for model development [4], considerations for making clinically useful models [5] and reporting prediction models [6]. However, there is currently no experiment-driven guidelines that inform researchers about how design choices to address loss to follow-up can impact prediction performance, so non-optimal design choices may commonly be leading to sub-optimal models. As a result, the developed prediction model may not perform as well as desired when applied in a real-world setting.

Binary classification models, such as logistic regression, aim to learn a mapping from the predictor space to a value between 0 and 1 that corresponds to the risk of the outcome occurring during the time-at-risk. These models are unable to incorporate loss to follow-up, so a choice is needed whether to (1) include patients who are lost to follow-up and assume whether they have the outcome prior to loss to follow-up is the ground truth or (2) exclude patients who are lost to follow-up. A third option, not considered in this paper, is to include all patients but apply imputation strategies to impute the missing outcomes in patients lost to follow-up. Cox regression aims to learn hazard rates per predictor and is a method that can include patients lost to follow-up. The baseline hazard function needs to be calculated if the Cox model is required to estimate outcome probability during the time-at-risk and this can often be complex. It is unknown whether it is preferable to use a survival model rather than a binary classifier when loss to follow-up is frequent. There have been various one-off comparisons between logistic regression and Cox regression for effect estimation [7, 8] and prediction [9, 10]. One key study compared various ways to deal with loss to follow-up for a single prediction question [11]. They developed a unique way of dealing with loss to follow-up by assigning weights based on survival probability to the datapoints used to train various machine learning models. Their results showed that the discrimination performance of the different methods was similar, but the calibration was better using their weighting approach. However, it is unclear to what extent these findings generalize to other prediction problems. There is currently no large-scale data-driven guideline based on empirical evidence that can help model developers decide the approach to take for prediction problems where patients are lost to follow-up.

We investigate the hypothesis that there is no impact on model performance estimates due to the strategy for addressing loss to follow-up when using a cohort design. We use synthetic data studies and an empirical assessment across 21 prediction questions using real world data to evaluate the impact of various simple strategies for dealing with loss to follow-up. These results will be used to provide best practice guidelines for dealing with loss to follow-up in healthcare prediction. We picked simple strategies that don’t require editing classifier software, so these strategies can be easily implemented by researchers.

## Methods

### Data

In this study we use data extracted from a US electronic healthcare record database Optum® de-identified Electronic Health Record Dataset **(**Optum EHR). This database contains medical records for 93,423,000 patients recorded between the years 2006–2018. The medical record data includes clinical information, inclusive of prescriptions as prescribed and administered, lab results, vital signs, body measurements, diagnoses, procedures, and information derived from clinical notes using Natural Language Processing (NLP).

The use of Optum EHR was reviewed by the New England Institutional Review Board (IRB) and were determined to be exempt from broad IRB approval.

### Strategies for developing patient-level prediction models with data containing loss to follow-up

We investigate four possible simple design choices for dealing with patients lost to follow-up, both with pros and cons, see Table 1. For all four designs a patient is labelled as having the outcome if she has the outcome recorded during the observed time-at-risk (the observed time-at-risk ends when a patient is lost to follow-up or the cohort study period ends).

**Table 1:** Candidate design choices for dealing with loss to follow-up

Design choice	Pros	Cons
1: Binary classification model using data that exclude all patients lost to follow-up [12, 13] (e.g., exclude any patient not observed for the full time-at-risk)	The labels are correct as we observed all the patients in the training data for the complete time-at-risk follow-up	We reduce the size of the training data (the longer the time-at-risk, the smaller the dataset)
		If the health outcome is often fatal, then we may exclude all or the majority of the patients who have the health outcome
		May limit model generalizability to only those who are healthy
2: Binary classification model using data that include all patients (including those lost to follow-up) [14] (e.g., include every patient in the cohort. A patient not observed for the full time-at-risk is included but their outcome is determined based on whether they experienced the outcome during the observed time-at-risk)	We do not compromise generalizability	Labels may be incorrect for those who are lost to follow-up (this noise may impact the model’s ability to learn)
	Larger sample size	
3: Binary classification model using data that exclude patients lost to follow-up unless they have the outcome prior to loss to follow-up [15] (e.g., only exclude patients not observed for the full time-at-risk if they did not have the outcome during the observed time-at-risk. This means patients with a partial time-at-risk who have the outcome during this time are still included)	The labels are correct	Generalizability may be compromised
	We include all outcomes	Outcome patients may be sicker as we can include those who die within time-at-risk but this is not possible for non-outcomes
	Do not lose outcomes when outcome is associated to death	
4: Cox model using data that includes all patients (including those lost to follow-up) [16] (e.g., include every patient, even those not observed for the full time-at-risk. The survival time is the minimum of time to end of observation, time to outcome or time-at-risk end (time to study period end from cohort index)	Method suitable for censored patients	Not intended for risk prediction, the main purpose is hazard rate calculation per predictor. Requires baseline hazard function for prediction
		Predict survival time (time before event) rather than risk of event
		Computationally more expensive

We used a least absolute shrinkage and selection operator (LASSO) logistic regression model as the classifier for solutions 1–3. For solution 4 we used a LASSO Cox regression model [17].

### Synthetic data study

We created partially synthetic data in two steps:

#### Step 1: Create partially synthetic data with no right censoring

We created a partially synthetic dataset using the following real-world prediction problem: ‘within patients who are pharmaceutically treated for depression, who will experience nausea within 3 years of the initial depression diagnosis?’ We extracted real world data on predictors, outcomes, and follow-up time from Optum EHR. The extracted data contained 86,360 randomly sampled patients in the target population (we sampled 100,000 but 13,640 patients had nausea prior to index and were excluded), of which 52,325 (60.5%) lacked complete 3-year time-at-risk follow-up. To create a dataset with complete follow-up, we trained a prediction model to predict nausea on this dataset and then applied it to the patients lost to follow-up to impute whether they had the outcome. For each patient lost to follow-up we drew a number from a uniform distribution X ~ U(0,1) and if this value was less than or equal to the predicted risk of the patient experiencing the outcome then the patient was labelled as an outcome patient, otherwise they were labelled as non-outcome. This resulted in 8944 patients lost to follow-up being labeled as having the outcome and 43,381 labeled as not having the outcome. For each patient with the outcome imputed, we also randomly selected the date at which they had the outcome by randomly picking uniformly between their start date and 3 years following. Full details of the method used to create the partially synthetic data are available in “Appendix 3”.

We chose to impute the outcome for patients lost to follow-up rather than restrict to patients who were not lost to follow-up due to potential bias issues. If the patients lost to follow-up were systematically different to the patients not lost to follow-up, then the results observed when analyzing the impact of loss to follow-up restricted to patients with complete follow-up may not generalize to the whole population.

#### Step 2: Simulating loss to follow-up

Starting with the partially synthetic dataset from step 1 that considers every patient to have complete follow-up, we then partition this set into 75% training data and 25% test data. We then simulate loss to follow-up in the training data based on either random selection or morbidity-based selection:

1. To simulate random loss to follow-up at a rate of *thres*% (*thres* in {10,20,30,40,50,70,90}) we draw from a uniform distribution per patient i, X1_i_ ~ U(0,1), and censor the ith patient if the number is less than the censoring rate X1_i_ < *thres/100* (e.g., if the censoring rate is *thres* = 10, then patients are censored if their randomly drawn number is 0.1 or less).
2. To simulate morbidity-based loss to follow-up at a rate of *thres*% we calculate each patient’s baseline Charlson comorbidity index score and then find the score where thres% of patients have a score equal or higher. We then consider all patients with that score or higher to be censored.

For patients who are identified as being lost to follow-up, we then simulate when they were lost. To simulate the date a patient is lost to follow-up, we uniformly picked the date during the 3-year follow-up (1095 days). For example, to simulate the date we draw a number from a uniform distribution, X2_j_ ~ U(0,1), per patient *j* and set their censored date as start_date_j_ + floor(1095*X2_j_) where start_date_j_ is the date patient j entered the target cohort. If a patient has the outcome at a date after their loss to follow-up date, then the outcome would have been observed after loss to follow-up, so we revise these patients to be labelled non-outcome patients. If the patient has the outcome on a date before the loss to follow-up date, then we would have seen the outcome prior to loss to follow-up, so they are still considered to be labelled as outcome patients.

**Fig. 1:**
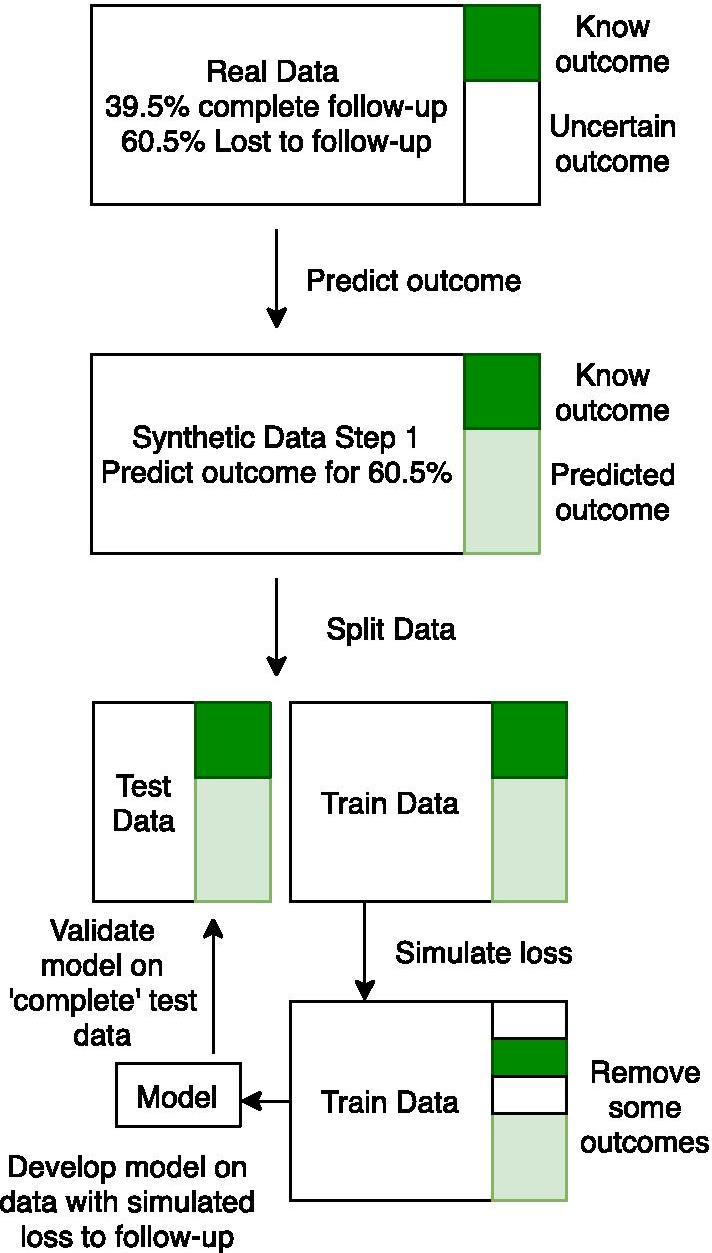
Creating the synthetic data and using it for model development and validation

We do not simulate loss to follow-up on the 25% test set, as this ‘silver standard’ is used to evaluate the impact of the four different solutions for developing patient-level prediction models in data containing loss to follow-up. The creation of the synthetic data is illustrated in Fig. 1.

### Empirical real-world data study

In addition to investigating the impact of dealing with loss to follow-up using a partially synthetic data set with ground truth labels, we repeated the investigation using real word data. For each simple loss to follow-up strategy we empirically investigate the performance when addressing 21 different prediction problems for two different follow-up periods (time-at-risk of 1 year and 3 years after index) using real world data. In a previous study we developed models to predict 21 different outcomes in a target population of pharmaceutically treated depressed patients [3]. For consistency, here we picked the same 21 prediction problems.

The target population of pharmaceutically treated depressed patients are defined as:

- Index rule defining the target population index dates:
- First condition record of major depressive disorder

Inclusion criteria:

- Antidepressant recorded within 30 days before to 30 days after the target population index date
- No history of psychosis
- No history of dementia
- No history of mania
- ≥ 365 days prior observation in the database
- ≥ 30 days post observation in the database

The 21 outcomes were: gastrointestinal hemorrhage, acute myocardial infarction, stroke, suicide and suicidal ideation, insomnia, diarrhea, nausea, hypothyroidism, constipation, seizure, delirium, alopecia, tinnitus, vertigo, hyponatremia, decreased libido, fracture, hypotension, acute liver injury and ventricular arrhythmia and sudden cardiac death. All definitions and logic used to define these outcomes are supplied in Additional file 1: Supplement A.

Real world labelled data were extracted from Optum EHR for each prediction problem. We created labels for each patient and time-at-risk (1-year and 3-years). For each prediction problem, the binary classifier outcome label was 1 if the patient had the outcome recorded during the time-at-risk following index and 0 otherwise. We did not impute any outcomes for patients lost to follow-up in the real-world data. The predictors were the presence of medical conditions and drugs that occurred prior to index or demographics at index. We created binary indicator variables for every condition and drug one or more of the target population had recorded prior to index. For example, if a patient had a record of type 1 diabetes prior to index, we could create a variable ‘type 1 diabetes any time prior’. Any patient who had type 1 diabetes recorded prior to index would have a value 1 for the variable ‘type 1 diabetes any time prior’ and any patient who did not have a type 1 diabetes record prior to index would have a value of 0. In total we extracted 204,186 variables.

We then partitioned the labelled data into 75% training set and 25% test set. The four design choices were each independently applied for each prediction problem and models were developed using the training data.

### Performance evaluation

We evaluate the models’ performances by calculating the area under the receiver operating characteristic curve (AUROC) on the test data with and without the patients lost to follow-up. An AUROC of 0.5 is equivalent to random guessing and an AUROC of 1 corresponds to perfect discrimination (able to identify the people who will develop the outcome at a specific risk threshold). The Cox regression AUROC was calculated using the exponential of the sum of the effect parameters multiplied by the covariate values (without the baseline hazard function).

## Results

### Partially synthetic data studies

The results of the analysis on the synthetic data are presented in Tables 2 and 3. In these results the ‘silver standard’ test data contained complete follow-up for each patient, but in the train data we simulated that *thres*% of patients were lost to follow-up. Table 2 corresponds to when loss to follow-up is randomly simulated, whereas Table 3 corresponds to when loss to follow-up was based on a patient’s health. If a patient with the outcome (when they had full follow-up) had a simulated loss to follow-up then two situations were possible i) the outcome date was before the date they were lost to follow-up (before loss to follow-up date) or ii) the outcome date was after the date they were lost to follow-up (after loss to follow-up). If the outcome date was after the simulated loss to follow-up date, then the patient’s label in the train data was set to non-outcome (noisy data). When loss to follow-up was random the solutions performed similarly in terms of discrimination (Table 2). When loss to follow-up was more common in sicker patients, more outcome patients were lost to follow-up and the solution ‘Logistic remove lost to follow-up non-outcomes’ performed worse in terms of discrimination on the test set (Table 3).

**Table 2:** AUROC results when predicting the simulated outcome within 3 years, when loss to follow-up is at random

Percentage censored (*thres*) (%)	Number in training Target Pop (64,770) censored	Training Outcome count (10,104) with loss to follow-up		Logistic keep lost to follow-up	Logistic remove lost to follow-up	Logistic remove lost to follow-up non-outcomes	Cox keep lost to follow-up
		Before loss to follow-up date	After loss to follow-up date	Test AUROC (train AUROC)			
~ 10	6532	434	586	0.690 (0.703)	0.690 (0.705)	0.693 (0.714)	0.690 (0.702)
~ 20	12,914	836	1201	0.690 (0.703)	0.690 (0.715)	0.692 (0.716)	0.689 (0.701)
~ 30	19,536	1218	1813	0.691 (0.714)	0.691 (0.713)	0.691 (0.718)	0.684 (0.700)
~ 40	26,002	1668	2440	0.692 (0.712)	0.686 (0.715)	0.691 (0.716)	0.688 (0.699)
~ 50	32,460	2140	3054	0.688 (0.699)	0.697 (0.714)	0.691 (0.717)	0.688 (0.698)
~ 70	45,401	2924	4216	0.687 (0.699)	0.678 (0.712)	0.688 (0.718)	0.686 (0.695)
~ 90	58,356	3766	5339	0.685 (0.699)	0.664 (0.715)	0.679 (0.721)	0.684 (0.695)

**Table 3:** AUROC results when predicting the simulated outcome within 3 years, when loss to follow-up is based on Charlson comorbidity index

Percentage censored (*thres*) (%)	Number in training Target Pop (64,770) censored	Training Outcome count (10,104) lost to follow-up		Logistic keep lost to follow-up	Logistic remove lost to follow-up	Logistic remove lost to follow-up non-outcomes	Cox keep lost to follow-up
		Before loss to follow-up date	After loss to follow-up date	Test AUROC (train AUROC)			
~ 10	6488	527	901	0.685 (0.697)	0.684 (0.702)	0.675 (0.735)	0.685 (0.693)
~ 20	12,946	1024	1606	0.680 (0.695)	0.683 (0.711)	0.654 (0.754)	0.684 (0.687)
~ 30	19,371	1422	2294	0.678 (0.692)	0.681 (0.710)	0.636 (0.778)	0.682 (0.680)
~ 40	25,834	1925	2847	0.677 (0.692)	0.679 (0.707)	0.621 (0.800)	0.682 (0.675)
~ 50	32,313	2289	3450	0.677 (0.692)	0.676 (0.706)	0.607 (0.837)	0.681 (0.671)
~ 70	45,271	2973	4387	0.681 (0.708)	0.671 (0.693)	0.592 (0.865)	0.678 (0.674)
~ 90	58,274	3726	5394	0.684 (0.714)	0.654 (0.723)	0.590 (0.916)	0.676 (0.689)

The calibration plots, see “Appendix 1”, show that the logistic models trained using data that excluded all patients lost to follow-up are generally well calibrated, but the other models were poorly calibrated when there was a high percentage of loss to follow-up (*thres* > 30%). The ‘keep all lost to follow-up’ LASSO logistic regression models appear to slightly underestimate the risk, whereas the ‘remove lost to follow-up non-outcomes’ solution substantially overestimated the risk. The miscalibration was worse as the number of patients lost to follow-up increased. Figure 10 in “Appendix 1” shows the development data outcome rates as a function of percentage of loss to follow-up for each simple solution. The calibration results are clearly explained by the trends in Fig. 10. The Cox regression requires the calculation of the baseline hazard function before it can be used to calculate the probability that a patient experiences the outcome during the time-at-risk period. The tool we used for LASSO Cox regression does not provide this function and calibration could not be calculated.

### Empirical real-world data studies

**Fig. 2:**
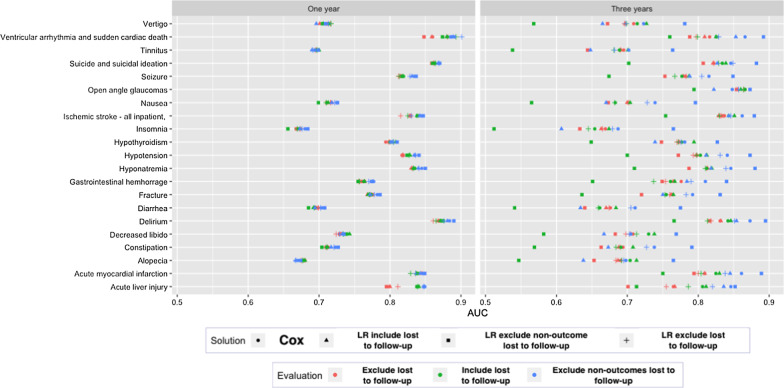
The test AUROC performances for four censoring solutions on three difference test sets across 21 prediction questions

The results of each solution when predicting the various outcomes within 1-year or 3-years of the initial treatment for depression across the three test datasets are presented in Fig. 2. The results are also available as Table 4 in “Appendix 2”.

Figure 2 shows the performance of the four solutions are similar when the time-at-risk is 1 year except when the outcome count is low (acute liver injury) or the outcome is associated to loss to follow-up (ventricular arrythmia and sudden cardiac death). The performance is more varied when the time-at-risk is 3 years. When the time-at-risk increases to 3 years, the LASSO logistic regression trained using data that removed the lost to follow-up non-outcome patients seems to consistently perform worse when evaluated on the data keeping all patients lost to follow-up or excluding all patients lost to follow-up.

#### Empirical results for 1 to 8-year time-at-risk

We highlight liver injury, because it is the rarest outcome, as well as suicide and suicidal ideation because it is likely associated to loss to follow-up (e.g., if the patient dies by suicide). For these two outcomes we compare the discrimination of the regularized logistic regression trained on data including lost to follow-up patients and the regularized Cox model for various time-at-risks. We trained the models on 75% of the data, including those who were lost to follow-up. To evaluate we used the test set containing 25% of the data, both when including all patients who were lost to follow-up (keep all) and when excluding all the patients who were lost to follow-up (remove all).

**Fig. 3:**
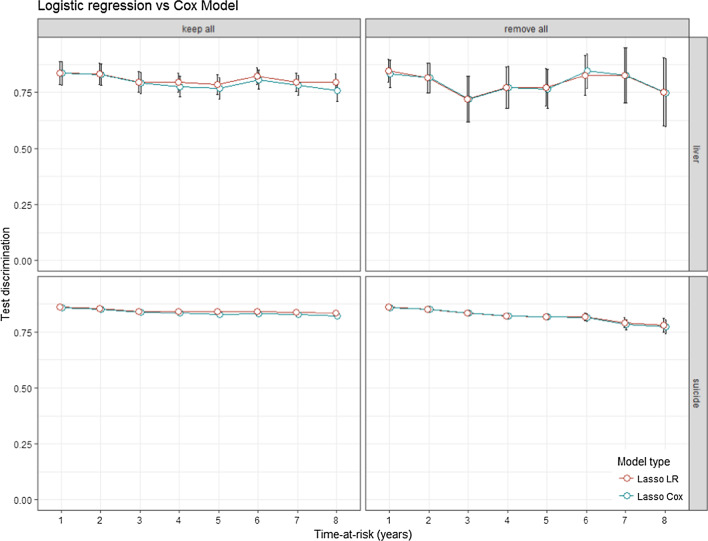
Comparing LASSO logistic regression and LASSO Cox regression both trained on data including patients lost to follow-up for time-at-risk periods between 1 and 8 years. The left-hand plots are the discrimination performance (AUROC) when evaluated on a test set that included patients lost to follow-up and the right-hand plots are the discrimination performance (AUROC) when evaluated on a test set that excluded patients lost to follow-up. The rows are the different outcomes (top row corresponds to the liver injury outcome models and bottom row corresponds to the suicide attempt and ideation outcome models)

**Fig. 10:**
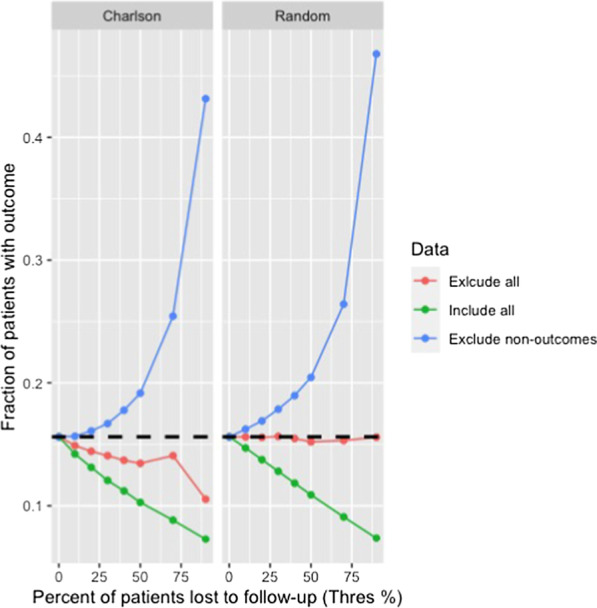
The percentage of the partially synthetic development data with the outcome based on the simple solution for addressing loss to follow-up as a function of the loss to follow-up amount. The black dashed line is the true percentage of patients with the outcome. The red line is the outcome % in the data when all patients lost to follow-up are excluded, green is the % in the data when all the patients lost to follow-up are included and blue is the data when all patients without the outcome lost to follow-up are excluded

Figure 3 shows that the discrimination performance was similar between a Cox regression model and a logistic regression model that used LASSO regularization and were trained using data that included patients lost to follow-up for the two prediction questions. As the time at risk increases the number of patients lost to follow-up increases, making the performance less certain in the test set that excluded patients lost to follow-up (larger confidence intervals on the right).

## Discussion

In this study we compared the performance of four different simple solutions to address loss to follow-up by using a partially synthetic dataset and 21 real world prediction questions. The simulation results suggest that when loss to follow-up is random the solution makes little impact on discrimination. However, the calibration was impacted when there was sufficient loss to follow-up, except in the cases of the models developed using data that excludes all patients lost to follow-up. When the loss to follow-up was simulated based on comorbidity, the models developed using data that excluded patients lost to follow-up unless they had the outcome during the time-at-risk prior to censoring had much worse discriminative performance than the other strategies and were poorly calibrated. The real-world problems predicting 1-year risk of various outcomes using different strategies to address loss to follow-up showed the strategy had little impact on discriminative performance. The 3-year real-world data models showed variability in the discrimination ability based on the strategies. This may have been because the 3-year time-at-risk has more patients that are lost to follow-up. In general, we found:

1. The binary classification models trained when excluding all patients lost to follow-up resulted in well calibrated models with good discriminative performance in both the random and comorbidity based simulated scenarios. This was observed even in the worst-case scenario where a large number of the most ill patients were lost to follow-up. This makes sense if the loss to follow-up is independent of the outcome, as the non-outcome and outcome patients should have an equal chance of being lost to follow-up. However, this is not a feasible solution if excluding patients lost to follow-up reduces the data size too much. In addition, the real-world data suggests this approach is problematic when the outcome is linked to loss to follow-up (see Fig. 3 ‘ventricular arrhythmia and sudden cardiac death’ and ‘acute myocardial infarction’ outcomes where the models trained using all the data outperformed the model trained using data that excluded patients lost to follow-up). Hypothetically, if the outcome was linked to death (e.g., acute myocardial infarction’), then excluding patients lost to follow-up (those who died due to acute myocardial infarction’), would result in a model that predicts surviving acute myocardial infarction attempt rather than all acute myocardial infarctions.
2. The binary classification models trained when including all patients lost to follow-up appear to have good discrimination but slightly under-estimate risk due to some of the patients with the outcome being misclassified as non-outcomes. LASSO logistic regression will be able to account for some noise but using a more noise-robust classifier may be preferable when loss to follow-up is common [18, 19]. The tolerance to small amounts of noise may explain why the discrimination performance appears to be generally unaffected when including noisy labels up to a certain quantity. However, not observing all patients for the complete time-at-risk results in less outcomes (as patients who may have had the outcome after being lost to follow-up are incorrectly labelled as non-outcomes) and this resulted in an under-estimation of risk. This is a limitation that must be highlighted if using this approach. It may be possible to recalibrate if the true outcome rate is known.
3. The survival models (LASSO Cox) trained when including all patients lost to follow-up appear to have good discrimination but are slower to train and require estimating the baseline hazard to calculate calibration. In this paper we found that the discrimination performance for the LASSO logistic regression and LASSO Cox models trained using data including lost to follow-up patients across various time at-risk periods, from 1 year up to 8 years, appear to be equivalent.
4. The binary classification models trained when excluding patients lost to follow-up who do not have the outcome prior may have high discrimination when tested on data with the same exclusion rules. However, these models appear to answer ‘what is my risk of having the outcome or being lost to follow-up’ (as only outcome patients lost to follow-up can be in the development data) and can perform poorly in terms of discrimination and calibration when answering the intended question ‘what is my risk of the outcome during time-at-risk’. For example, the models often over-estimated risk. This makes sense as censoring the non-outcomes lost to follow-up results in a higher outcome % (as the outcome count is the same but the study population reduces) in the development data, causing calibration issues. For this strategy, the train set discriminative performance was generally higher than the other solutions, but the test set discriminative performance was lower. This indicates the model is often not transportable to patients who were lost to follow-up without experiencing the outcome during the time-at-risk. This makes sense, as sicker patients (who are likely to be lost to follow-up due to death) are only included in the development data if they have the outcome before censoring, so the outcome patients in these data will be artificially sicker. This can cause generalizability issues.

In summary, if a researcher needs to pick a simple strategy to address loss to follow-up when using a cohort design, then he should avoid excluding patients who are lost to follow-up without experiencing the outcome during the time-at-risk but including patients who are lost to follow-up after experiencing the outcome during the time-at-risk. This strategy consistently led to poorly calibrated models that may not answer the intended question. If experiencing the outcome is likely to increase the chance of being lost to follow-up or the data are small, then excluding patients lost to follow-up is likely to be detrimental in terms of discrimination. In this case, training a model using slightly noisy data that includes patients lost to follow-up is preferable. However, this is likely to lead to slightly miscalibrated models. Recalibration should be attempted if the true outcome rate is known or the calibration issue should be highlighted as a potential limitation. Based on our simulation and empirical evaluation, it is our opinion that:

1. The LASSO Cox model does not appear to be better than training a LASSO logistic regression model, in terms of discrimination, with training data that includes all patients lost to follow-up up to the 8-year time-at-risk investigated. Future work should investigate whether using a LASSO Cox model can lead to better calibration.
2. Training a model using data that removed patients lost to follow-up who do not have the outcome but kept those with the outcome can bias a model and lead to models that overestimate risk.
3. Evaluating a model on data that removed patients lost to follow-up who do not have the outcome but kept those with the outcome can lead to optimistic performance estimates.
4. If the loss to follow-up is associated with the outcome (i.e., the outcome can cause death) or the outcome count is low then training a model on data where patients lost to follow-up are removed could limit performance.
5. Training models using data that include patients lost to follow-up can lead to miscalibrated models as the outcome percentage in the data is diluted.

As best practices we propose that researchers (1) develop models using data that includes patients that are lost to follow-up as this is less likely to lead to biased models (but use noise tolerant binary classifiers or survival models), (2) perform recalibration if possible to address the miscalibration issue and (3) evaluate the model performance on test data that includes patients that are lost to follow-up but also evaluate the model performance on test data that excludes patients that are lost to follow-up to gain more insight into the true model performance.

A strength of this study is that we were able to empirically evaluate the impact of various solutions to deal with loss to follow-up at scale. In this study we developed 4 models in 2 time-at-risk periods for 21 outcomes, so 168 models in total. In future work it may be useful to expand this further and evaluate whether the results hold across more datasets and prediction questions. In addition, it would be useful to investigate the performance on external datasets to see which solutions are more generalizable. Our results for the partially synthetic study are dependent on the technique we used to impute the outcome labels and the methods used to simulate loss to follow-up. A limitation of our partially synthetic study is that we made certain assumption such as that the loss to follow-up date was uniform between the time-at-risk period, whereas in reality you may find censoring more common at the start or end of the follow-up. In addition, for the Charlson comorbidity-based simulation we decided to investigate the worst-case scenario, where the sickest patients were lost to follow-up. Therefore, our results using the partially synthetic data may be due to the imputation and simulation designs. In future it may be useful to study more simulation scenarios to gain a greater theoretical understanding, especially for scenarios where the outcome is associated to loss to follow-up. There have been numerous methods to address missing outcome data [20, 21] and in future work it would be interesting to see whether our partially synthetic results hold when using different techniques to create the partially synthetic data. However, our empirical results used real world data that would capture any data complexities such as loss to follow-up distribution, so these are more informative. Although, we only tested the solutions on 21 real world prediction questions, and it is not possible to know whether our results would generalize to all prediction questions.

The problem of loss to follow-up in cohort studies is effectively a missing outcome data problem. In this study we did not consider using imputation methods to address the outcome missingness, instead we focused on simple methods using noisy labels, complete case analysis or survival models that can handle the missingness. There are a range of imputation techniques that are often used in clinical trial studies with missing outcome data [20], however these generally make assumptions about the missingness mechanism that can be impossible to confirm. In addition, studies have shown that misspecified outcome imputation models can cause bias in relative risk estimates [22], so bias issues may also occur when using imputation to address loss to follow-up in prognostic model development. In future work it would be interesting to further investigate and compare whether methods to impute the missing outcomes could be used as an alternative strategy for addressing loss to follow-up. In addition, there are other solutions available for addressing loss to follow-up that were not investigated. For example, patients lost to follow-up could have a lower weight assigned when calculating the model performance, so they have less impact. However, we selected the four solutions investigated in this paper due to their simplicity so they could be widely implemented without advanced knowledge of machine learning or programming, as this is likely to limit a solution’s utility.

This is the first study to empirically evaluate simple design choice for dealing with loss to follow-up data in prediction model development at scale and our results can now be used to guide other researchers. It is important to note that this study does show superiority of any method, but it does highlight the pitfalls of some simple approaches to censored data and illustrates the trade-off between noise and bias.

## Conclusion

We compared four different techniques that can be used to address the issue of loss to follow-up in prediction model development. Our results suggest that using training data that removes patients who are lost to follow-up who do not have the outcome but keeps patients lost to follow-up who have the outcome can lead to biased models. Based on this research it appears that it is best to develop models using data that includes patients that are lost to follow-up. However, recalibration is likely to be required as this strategy appears to result in models that under-estimate risk.

## Supplementary Information

**Additional file 1.** The logical definitions for the 21 outcomes used in the empirical real-world data studies.

## Figures and Tables

**Fig. 4:**
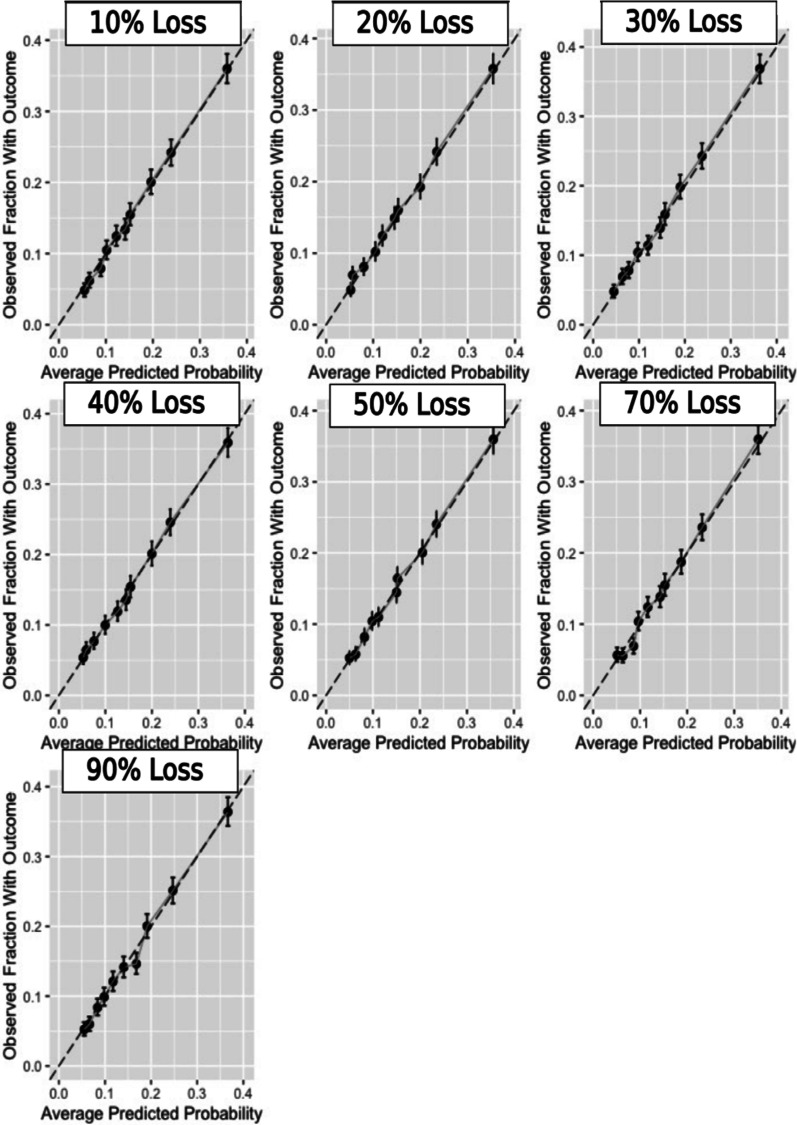
Calibration of model trained on data excluding patients with loss to follow-up after simulating N% of patients were lost to follow-up randomly (MCAR)

**Fig. 5:**
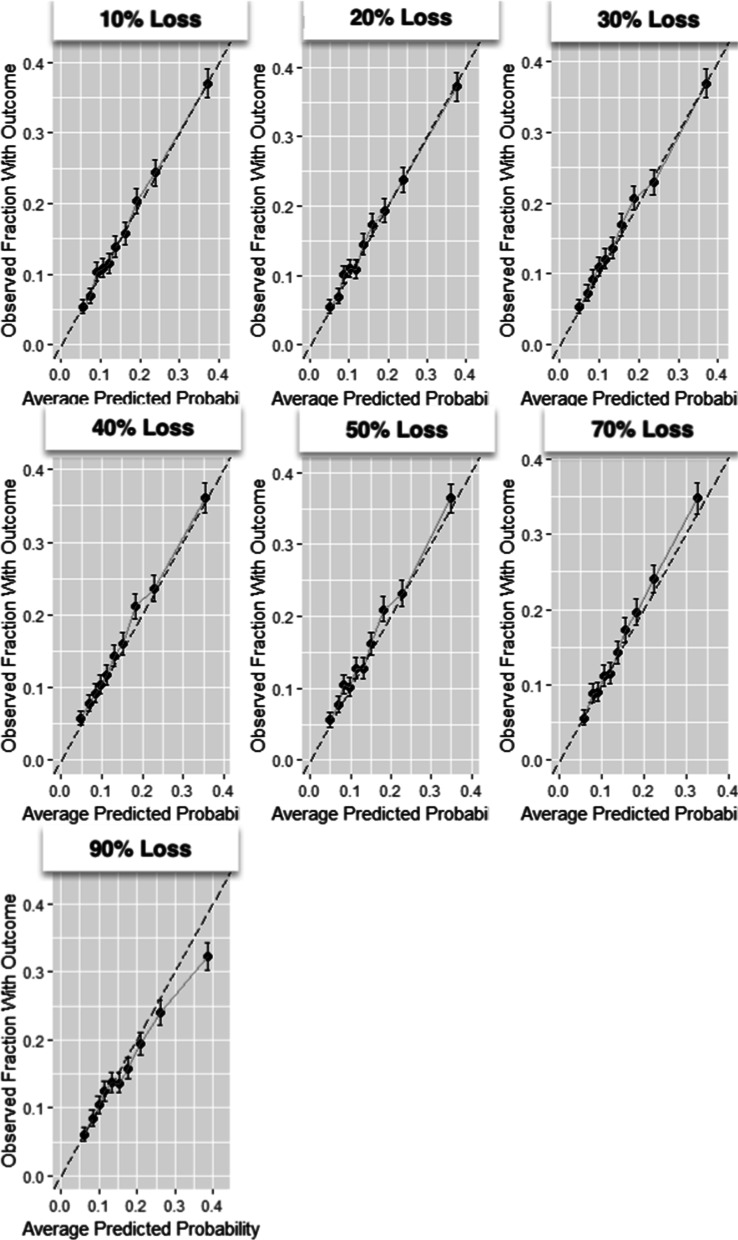
Calibration of model trained on data excluding patients with loss to follow-up after simulating N% of patient were lost to follow-up based on Charlson's comorbidity index

**Fig. 6:**
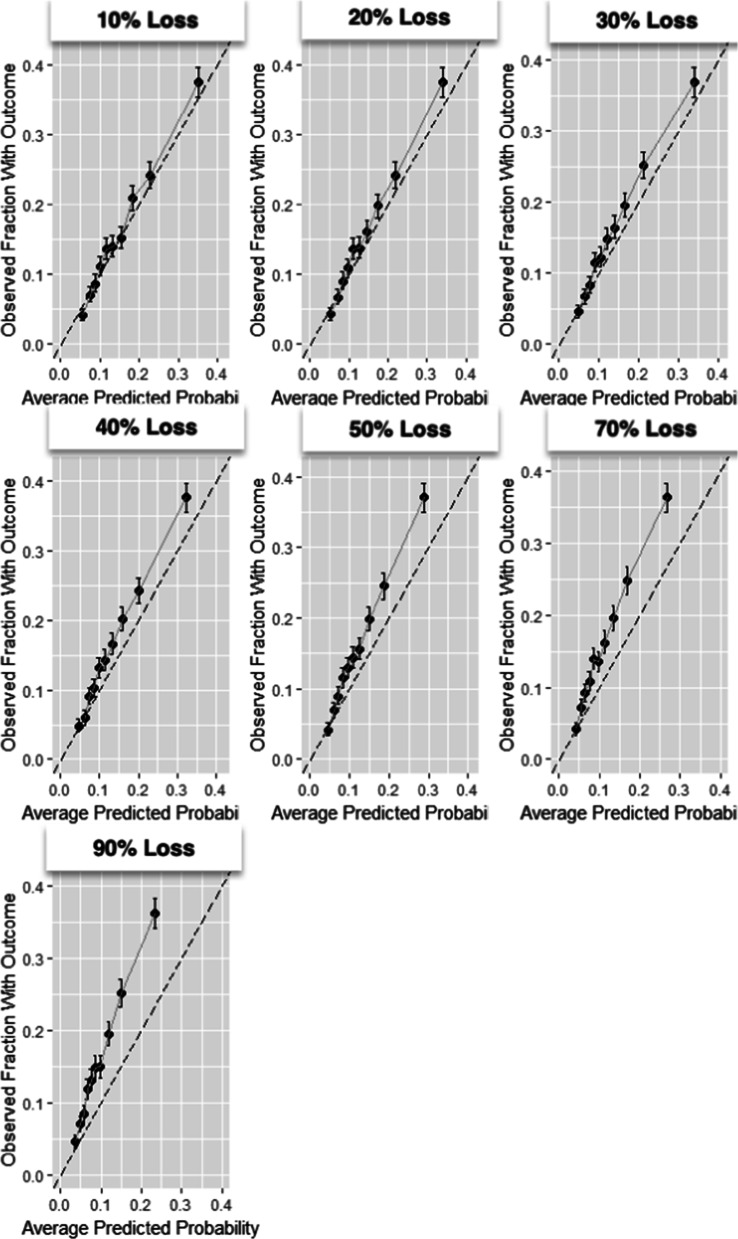
Calibration of model trained on data including patients with loss to follow-up after simulating N% of patient were lost to follow-up randomly (MCAR)

**Fig. 7:**
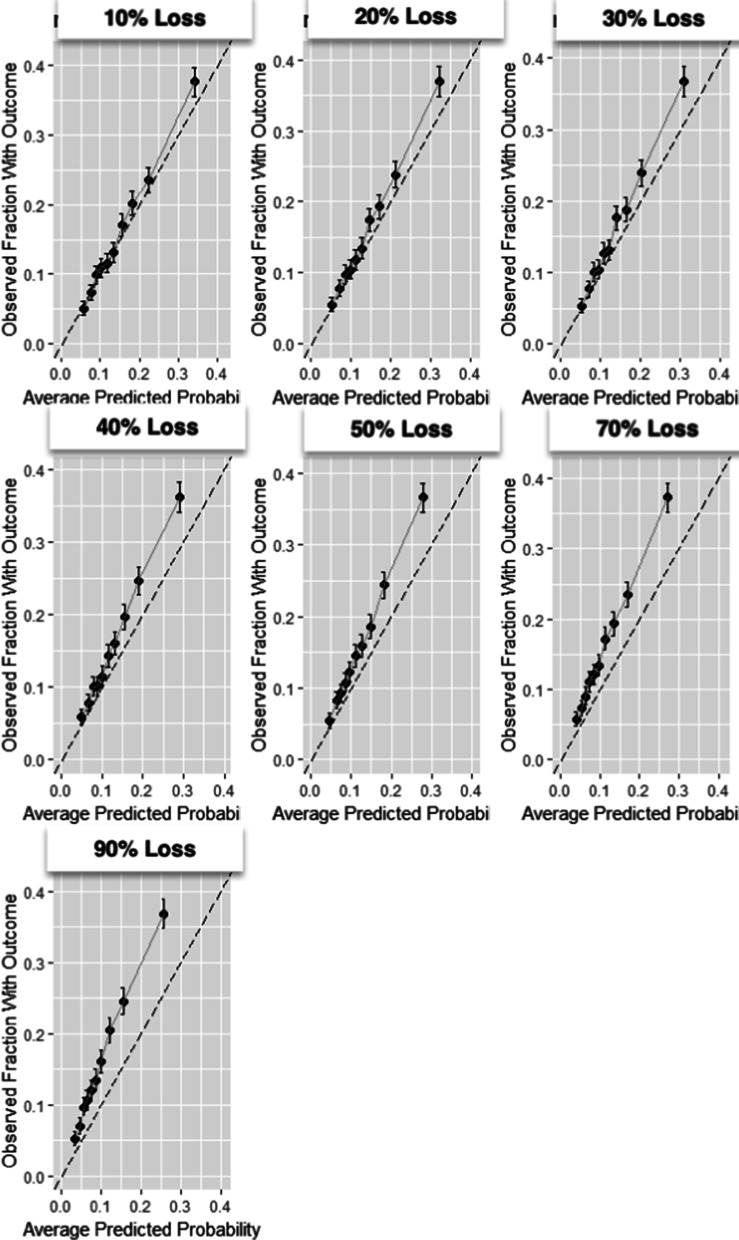
Calibration of model trained on data including patients with loss to follow-up after simulating N% of patients were lost to follow-up based on Charlson's comorbidity index

**Fig. 8:**
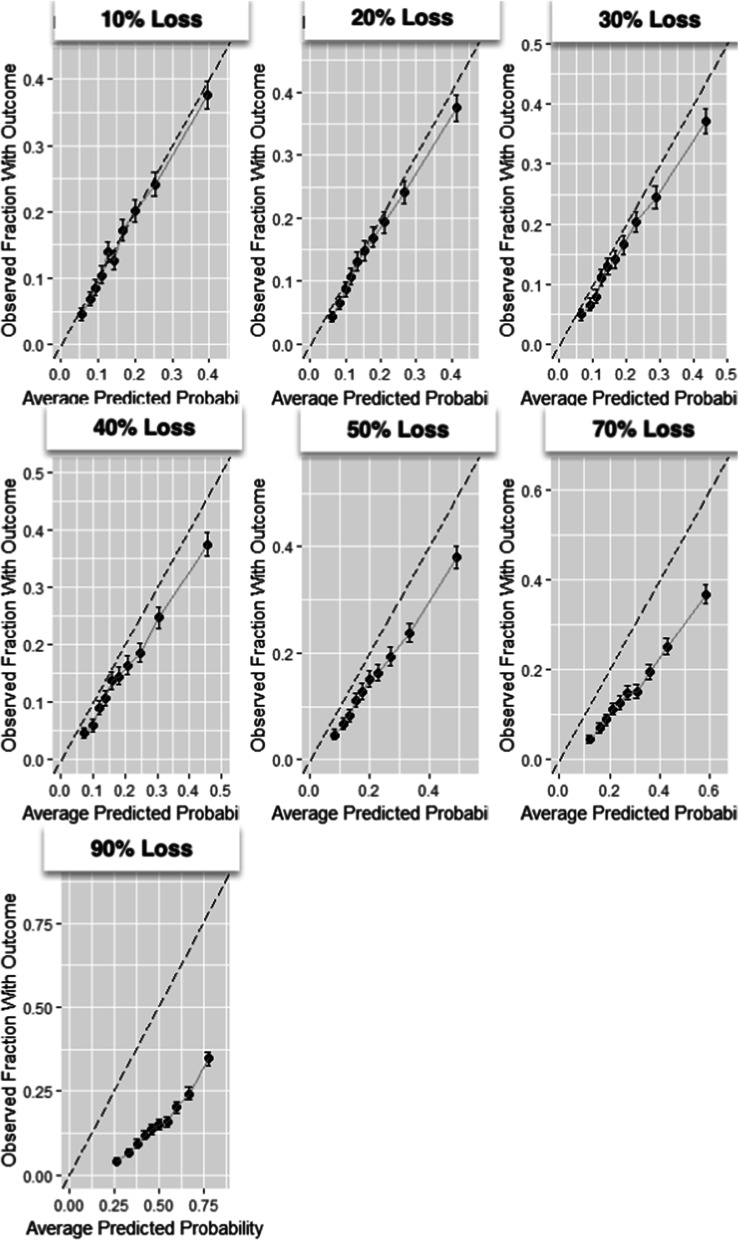
Calibration of model trained on data excluding patients with loss to follow-up if they had no outcome during the observed time-at-risk after simulating N% of patients were lost to follow-up randomly (MCAR)

**Fig. 9:**
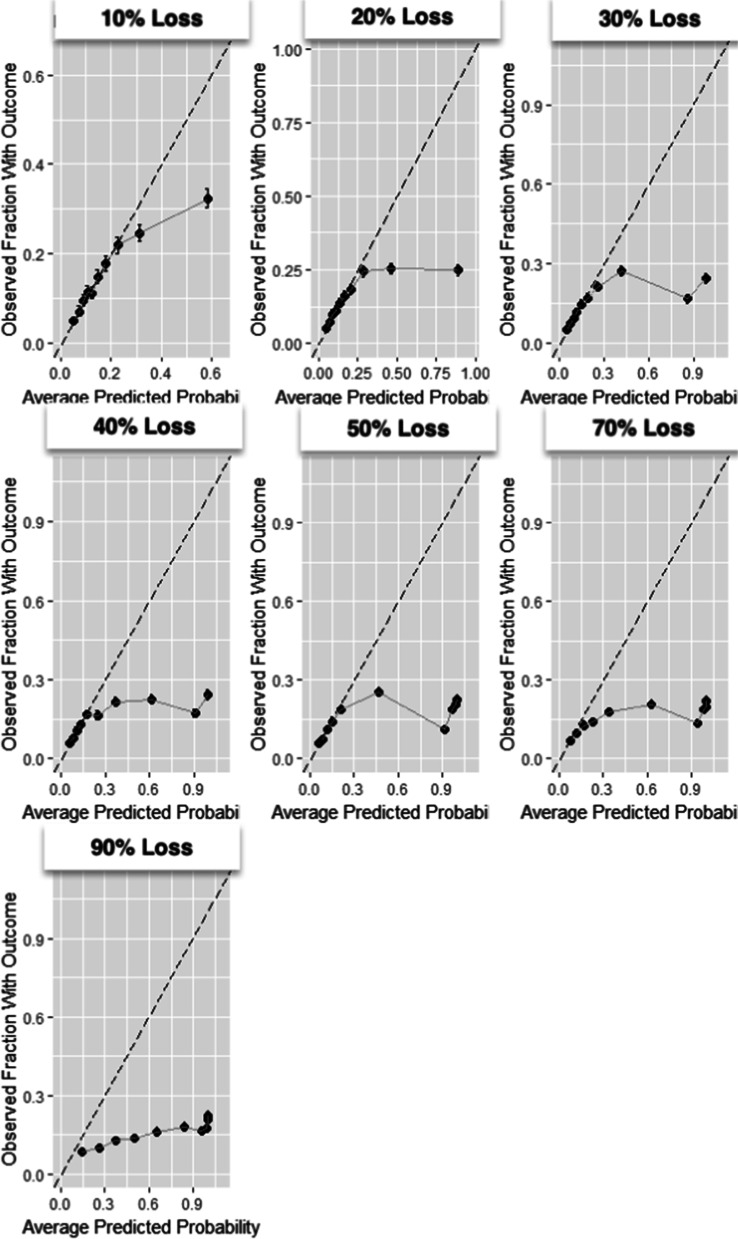
Calibration of model trained on data excluding patients with loss to follow-up if they had no outcome during observed time-at-risk after simulating N% of patients were lost to follow-up based on Charlson’s comorbidity index

## Data Availability

The Optum EHR data that support the findings of this study are available from Optum (contact at: https://www.optum.com/business/solutions/life-sciences/explore-data/advanced-analytics/ehr-data.html) but restrictions apply to the availability of these data, which were used under license for the current study, and so are not publicly available.

## Appendix 1

### Calibration plots

Calibration of lasso logistic regression models trained on data that excluded all patients lost to follow-up that were applied to test data with no loss to follow-up on simulated data. 10% loss corresponds to simulating that 10% of the target population leave during the follow-up, whereas 50% corresponds to 50% of the target population leaving during follow-up;

Calibration of lasso logistic regression models trained on data that included all patients lost to follow-up that were applied to test data with no loss to follow-up on simulated data:

Calibration of lasso logistic regression models trained on data that excluded all non-outcome patients lost to follow-up that were applied to test data with no loss to follow-up on simulated data (MCAR left, Charlson right).

### Outcome percent in development data

See Figs. 4, 5, 6, 7, 8, 9 and 10 .

## Appendix 2

See Tables 4 and 5.

**Table 4:** Full results tables

Outcome (outcome count with partial time-at-risk/ outcome count with full time-at-risk)	Solution	Test Data 1: All lost to follow-up patients removed (~ 97,500)	Test Data 2: All lost to follow-up patients included (~ 125,000)	Test Data 3: All non-outcome lost to follow-up patients are removed (~ 97,500)
Acute liver injury incident event (59/33)	Logistic remove lost to follow-up	0.811 (0.732–0.891)	0.839 (0.782–0.896)	0.848 (0.791–0.904)
	Logistic keep lost to follow-up	0.8 (0.709–0.89)	0.841 (0.779–0.902)	0.848 (0.787–0.909)
	Logistic remove non-outcomes lost to follow-up	0.795 (0.702–0.887)	0.838 (0.776–0.9)	0.848 (0.787–0.91)
	Cox keep lost to follow-up	0.799 (0.71–0.889)	0.84 (0.779–0.902)	0.849 (0.788–0.91)
Ventricular arrhythmia and sudden cardiac death incident event (68/38)	Logistic remove lost to follow-up	0.881 (0.825–0.938)	0.893 (0.857–0.93)	0.901 (0.866–0.937)
	Logistic keep lost to follow-up	0.859 (0.79–0.928)	0.88 (0.837–0.923)	0.889 (0.847–0.932)
	Logistic remove non-outcomes lost to follow-up	0.848 (0.77–0.925)	0.874 (0.827–0.921)	0.886 (0.84–0.932)
	Cox keep lost to follow-up	0.86 (0.791–0.929)	0.881 (0.837–0.924)	0.891 (0.848–0.933)
Ischemic stroke—all inpatient, incident event (95/73)	Logistic remove lost to follow-up	0.815 (0.771–0.859)	0.825 (0.789–0.861)	0.83 (0.794–0.866)
	Logistic keep lost to follow-up	0.828 (0.787–0.869)	0.838 (0.804–0.871)	0.842 (0.809–0.876)
	Logistic remove non-outcomes lost to follow-up	0.829 (0.788–0.869)	0.839 (0.806–0.872)	0.847 (0.814–0.879)
	Cox keep lost to follow-up	0.828 (0.787–0.869)	0.838 (0.805–0.871)	0.844 (0.811–0.877)
Acute myocardial infarction incident event (137/110)	Logistic remove lost to follow-up	0.836 (0.799–0.873)	0.829 (0.797–0.861)	0.836 (0.805–0.868)
	Logistic keep lost to follow-up	0.845 (0.81–0.88)	0.837 (0.807–0.867)	0.843 (0.813–0.873)
	Logistic remove non-outcomes lost to follow-up	0.849 (0.814–0.884)	0.839 (0.809–0.869)	0.849 (0.82–0.879)
	Cox keep lost to follow-up	0.847 (0.813–0.882)	0.838 (0.808–0.868)	0.846 (0.816–0.875)
Delirium incident event (215/157)	Logistic remove lost to follow-up	0.861 (0.835–0.887)	0.865 (0.844–0.886)	0.876 (0.855–0.896)
	Logistic keep lost to follow-up	0.868 (0.843–0.892)	0.871 (0.851–0.892)	0.881 (0.861–0.901)
	Logistic remove non-outcomes lost to follow-up	0.87 (0.847–0.893)	0.875 (0.856–0.894)	0.89 (0.872–0.908)
	Cox keep lost to follow-up	0.869 (0.844–0.893)	0.872 (0.852–0.892)	0.884 (0.865–0.903)
Gastrointestinal hemorrhage incident event (225/186)	Logistic remove lost to follow-up	0.756 (0.721–0.792)	0.757 (0.726–0.789)	0.77 (0.739–0.801)
	Logistic keep lost to follow-up	0.764 (0.731–0.798)	0.764 (0.734–0.794)	0.776 (0.746–0.806)
	Logistic remove non-outcomes lost to follow-up	0.76 (0.726–0.793)	0.755 (0.724–0.785)	0.774 (0.744–0.804)
	Cox keep lost to follow-up	0.764 (0.731–0.798)	0.763 (0.733–0.793)	0.777 (0.748–0.807)
Decreased libido incident event (291/257)	Logistic remove lost to follow-up	0.724 (0.695–0.753)	0.734 (0.707–0.761)	0.729 (0.701–0.756)
	Logistic keep lost to follow-up	0.731 (0.702–0.759)	0.743 (0.716–0.769)	0.733 (0.705–0.76)
	Logistic remove non-outcomes lost to follow-up	0.729 (0.701–0.758)	0.737 (0.711–0.764)	0.735 (0.709–0.762)
	Cox keep lost to follow-up	0.729 (0.7–0.758)	0.74 (0.713–0.767)	0.734 (0.707–0.761)
Seizure incident event (408/312)	Logistic remove lost to follow-up	0.812 (0.79–0.834)	0.813 (0.794–0.832)	0.828 (0.81–0.847)
	Logistic keep lost to follow-up	0.815 (0.793–0.837)	0.818 (0.799–0.837)	0.831 (0.812–0.849)
	Logistic remove non-outcomes lost to follow-up	0.816 (0.794–0.838)	0.817 (0.798–0.836)	0.837 (0.818–0.855)
	Cox keep lost to follow-up	0.816 (0.794–0.838)	0.819 (0.8–0.837)	0.834 (0.816–0.852)
Alopecia incident event (590/527)	Logistic remove lost to follow-up	0.675 (0.654–0.696)	0.677 (0.657–0.697)	0.667 (0.646–0.687)
	Logistic keep lost to follow-up	0.679 (0.658–0.7)	0.68 (0.661–0.7)	0.667 (0.647–0.687)
	Logistic remove non-outcomes lost to follow-up	0.679 (0.658–0.7)	0.676 (0.656–0.695)	0.674 (0.654–0.694)
	Cox keep lost to follow-up	0.68 (0.659–0.701)	0.679 (0.659–0.699)	0.67 (0.65–0.69)
Tinnitus incident event (663/582)	Logistic remove lost to follow-up	0.695 (0.674–0.716)	0.697 (0.677–0.716)	0.691 (0.671–0.711)
	Logistic keep lost to follow-up	0.696 (0.675–0.717)	0.7 (0.681–0.72)	0.69 (0.67–0.71)
	Logistic remove non-outcomes lost to follow-up	0.696 (0.676–0.717)	0.695 (0.675–0.714)	0.699 (0.68–0.719)
	Cox keep lost to follow-up	0.697 (0.676–0.718)	0.699 (0.68–0.719)	0.694 (0.674–0.714)
Vertigo incident event (785/708)	Logistic remove lost to follow-up	0.716 (0.698–0.735)	0.717 (0.7–0.735)	0.713 (0.696–0.731)
	Logistic keep lost to follow-up	0.701 (0.682–0.72)	0.706 (0.688–0.724)	0.696 (0.678–0.714)
	Logistic remove non-outcomes lost to follow-up	0.711 (0.692–0.73)	0.705 (0.687–0.723)	0.709 (0.69–0.727)
	Cox keep lost to follow-up	0.712 (0.694–0.731)	0.714 (0.696–0.732)	0.71 (0.692–0.728)
Fracture incident event (1050/843)	Logistic remove lost to follow-up	0.772 (0.756–0.788)	0.771	0.778
	Logistic keep lost to follow-up	0.768 (0.752–0.785)	0.769	0.772
	Logistic remove non-outcomes lost to follow-up	0.775 (0.759–0.792)	0.772	0.786
	Cox keep lost to follow-up	0.775 (0.759–0.792)	0.774	0.782
Hyponatremia incident event (1683/1258)	Logistic remove lost to follow-up	0.831	0.833	0.841
	Logistic keep lost to follow-up	0.83	0.833	0.84
	Logistic remove non-outcomes lost to follow-up	0.833	0.834	0.85
	Cox keep lost to follow-up	0.833	0.835	0.845
Suicide and suicidal ideation incident event (2230/1726)	Logistic remove lost to follow-up	0.862	0.864	0.869
	Logistic keep lost to follow-up	0.861	0.864	0.868
	Logistic remove non-outcomes lost to follow-up	0.859	0.86	0.87
	Cox keep lost to follow-up	0.861	0.863	0.869
Hypothyroidism incident event (2259/1930)	Logistic remove lost to follow-up	0.799	0.805	0.805
	Logistic keep lost to follow-up	0.8	0.808	0.803
	Logistic remove non-outcomes lost to follow-up	0.799	0.802	0.81
	Cox keep lost to follow-up	0.794	0.8	0.801
Hypotension incident event (2462/1856)	Logistic remove lost to follow-up	0.818	0.827	0.836
	Logistic keep lost to follow-up	0.818	0.828	0.834
	Logistic remove non-outcomes lost to follow-up	0.818	0.824	0.841
	Cox keep lost to follow-up	0.817	0.825	0.835
Constipation incident event (4089/3381)	Logistic remove lost to follow-up	0.712	0.71	0.721
	Logistic keep lost to follow-up	0.712	0.712	0.717
	Logistic remove non-outcomes lost to follow-up	0.71	0.704	0.727
	Cox keep lost to follow-up	0.713	0.711	0.723
Diarrhea incident event (4687/3957)	Logistic remove lost to follow-up	0.699	0.693	0.702
	Logistic keep lost to follow-up	0.695	0.692	0.694
	Logistic remove non-outcomes lost to follow-up	0.696	0.685	0.708
	Cox keep lost to follow-up	0.699	0.694	0.704
Insomnia incident event (5843/4960)	Logistic remove lost to follow-up	0.673	0.669	0.676
	Logistic keep lost to follow-up	0.673	0.673	0.673
	Logistic remove non-outcomes lost to follow-up	0.668	0.656	0.684
	Cox keep lost to follow-up	0.674	0.671	0.68
Nausea incident event (6003/4997)	Logistic remove lost to follow-up	0.716	0.71	0.722
	Logistic keep lost to follow-up	0.717	0.713	0.718
	Logistic remove non-outcomes lost to follow-up	0.711	0.699	0.726
	Cox keep lost to follow-up	0.718	0.711	0.725

**Table 5:** AUROC results when predicting the outcomes within 3 years of the treatment for depression

Outcome (outcome count with partial time-at-risk/ outcome count with full time-at-risk)	Solution	Test Data 1: All patients with partial time-at-risk are removed (~ 47,000)	Test Data 2: All patients with partial time-at-risk are included (~ 125,000)	Test Data 3: All non-outcome patients with partial time-at-risk are removed
Acute liver injury incident event (107/43)	Logistic remove lost to follow-up	0.755 (0.67–0.84)	0.786 (0.74–0.831)	0.82 (0.776–0.864)
	Logistic keep lost to follow-up	0.767 (0.683–0.851)	0.811 (0.765–0.858)	0.836 (0.79–0.882)
	Logistic remove non-outcomes lost to follow-up	0.701 (0.603–0.8)	0.713 (0.661–0.765)	0.852 (0.804–0.901)
	Cox keep lost to follow-up	0.765 (0.681–0.849)	0.806 (0.759–0.853)	0.846 (0.802–0.89)
Ventricular arrhythmia and sudden cardiac death incident event (126/32)	Logistic remove lost to follow-up	0.797 (0.721–0.874)	0.799 (0.762–0.837)	0.829 (0.792–0.865)
	Logistic keep lost to follow-up	0.809 (0.721–0.897)	0.825 (0.787–0.862)	0.853 (0.816–0.891)
	Logistic remove non-outcomes lost to follow-up	0.788 (0.706–0.871)	0.76 (0.72–0.801)	0.892 (0.859–0.925)
	Cox keep lost to follow-up	0.816 (0.731–0.902)	0.826 (0.788–0.864)	0.866 (0.83–0.902)
Ischemic stroke—all inpatient, incident event (185/94)	Logistic remove lost to follow-up	0.832 (0.793–0.871)	0.83 (0.805–0.854)	0.846 (0.822–0.87)
	Logistic keep lost to follow-up	0.829 (0.791–0.866)	0.851 (0.829–0.874)	0.843 (0.819–0.866)
	Logistic remove non-outcomes lost to follow-up	0.834 (0.798–0.87)	0.754 (0.727–0.781)	0.879 (0.857–0.902)
	Cox keep lost to follow-up	0.837 (0.799–0.874)	0.843 (0.82–0.866)	0.862 (0.839–0.885)
Acute myocardial infarction incident event (273/111)	Logistic remove lost to follow-up	0.8 (0.759–0.841)	0.804 (0.78–0.829)	0.838 (0.815–0.861)
	Logistic keep lost to follow-up	0.809 (0.768–0.85)	0.83 (0.806–0.853)	0.845 (0.822–0.869)
	Logistic remove non-outcomes lost to follow-up	0.794 (0.754–0.834)	0.75 (0.725–0.775)	0.889 (0.867–0.911)
	Cox keep lost to follow-up	0.809 (0.768–0.849)	0.825 (0.801–0.849)	0.861 (0.838–0.883)
Delirium incident event (359/148)	Logistic remove lost to follow-up	0.818 (0.786–0.85)	0.813 (0.793–0.833)	0.851 (0.833–0.87)
	Logistic keep lost to follow-up	0.832 (0.801–0.863)	0.846 (0.828–0.864)	0.854 (0.836–0.873)
	Logistic remove non-outcomes lost to follow-up	0.816 (0.784–0.847)	0.766 (0.745–0.787)	0.895 (0.879–0.912)
	Cox keep lost to follow-up	0.831 (0.801–0.862)	0.842 (0.824–0.86)	0.873 (0.856–0.89)
Gastrointestinal hemhorrage incident event (430/210)	Logistic remove lost to follow-up	0.754 (0.721–0.787)	0.737 (0.714–0.76)	0.79 (0.768–0.811)
	Logistic keep lost to follow-up	0.765 (0.732–0.798)	0.767 (0.745–0.789)	0.784 (0.763–0.806)
	Logistic remove non-outcomes lost to follow-up	0.749 (0.716–0.782)	0.651 (0.627–0.674)	0.84 (0.819–0.861)
	Cox keep lost to follow-up	0.776 (0.745–0.808)	0.761 (0.739–0.783)	0.81 (0.789–0.831)
Decreased libido incident event (643/391)	Logistic remove lost to follow-up	0.698 (0.674–0.722)	0.713 (0.695–0.732)	0.703 (0.684–0.722)
	Logistic keep lost to follow-up	0.705 (0.682–0.729)	0.738 (0.72–0.755)	0.667 (0.646–0.687)
	Logistic remove non-outcomes lost to follow-up	0.683 (0.659–0.708)	0.582 (0.563–0.601)	0.769 (0.751–0.788)
	Cox keep lost to follow-up	0.708 (0.685–0.731)	0.73 (0.712–0.747)	0.705 (0.687–0.724)
Seizure incident event (712/313)	Logistic remove lost to follow-up	0.783 (0.757–0.809)	0.767 (0.75–0.784)	0.805 (0.789–0.822)
	Logistic keep lost to follow-up	0.779 (0.753–0.806)	0.787 (0.77–0.804)	0.788 (0.77–0.806)
	Logistic remove non-outcomes lost to follow-up	0.753 (0.725–0.78)	0.674 (0.656–0.692)	0.849 (0.833–0.865)
	Cox keep lost to follow-up	0.782 (0.756–0.808)	0.777 (0.76–0.794)	0.815 (0.798–0.832)
Alopecia incident event (1268/690)	Logistic remove lost to follow-up	0.684 (0.666–0.702)	0.692	0.695
	Logistic keep lost to follow-up	0.687 (0.669–0.706)	0.713	0.638
	Logistic remove non-outcomes lost to follow-up	0.653 (0.633–0.672)	0.547	0.765
	Cox keep lost to follow-up	0.691 (0.673–0.71)	0.704	0.698
Tinnitus incident event (1419/760)	Logistic remove lost to follow-up	0.69 (0.671–0.709)	0.681	0.682
	Logistic keep lost to follow-up	0.691 (0.672–0.71)	0.701	0.648
	Logistic remove non-outcomes lost to follow-up	0.644 (0.624–0.663)	0.538	0.764
	Cox keep lost to follow-up	0.695 (0.676–0.713)	0.689	0.702
Vertigo incident event (1663/911)	Logistic remove lost to follow-up	0.696 (0.679–0.713)	0.699	0.698
	Logistic keep lost to follow-up	0.697 (0.68–0.714)	0.727	0.665
	Logistic remove non-outcomes lost to follow-up	0.672 (0.655–0.69)	0.568	0.781
	Cox keep lost to follow-up	0.709 (0.693–0.726)	0.714	0.723
Fracture incident event (2222/1002)	Logistic remove lost to follow-up	0.76	0.753	0.783
	Logistic keep lost to follow-up	0.762	0.765	0.75
	Logistic remove non-outcomes lost to follow-up	0.72	0.636	0.831
	Cox keep lost to follow-up	0.764	0.755	0.792
Hyponatremia incident event (3032/1281)	Logistic remove lost to follow-up	0.812	0.81	0.839
	Logistic keep lost to follow-up	0.813	0.819	0.818
	Logistic remove non-outcomes lost to follow-up	0.787	0.71	0.88
	Cox keep lost to follow-up	0.812	0.812	0.846
Suicide and suicidal ideation incident event (3382/1549)	Logistic remove lost to follow-up	0.822	0.829	0.849
	Logistic keep lost to follow-up	0.821	0.839	0.827
	Logistic remove non-outcomes lost to follow-up	0.807	0.702	0.882
	Cox keep lost to follow-up	0.822	0.834	0.846
Hypothyroidism incident event (4119/2330)	Logistic remove lost to follow-up	0.77	0.772	0.776
	Logistic keep lost to follow-up	0.773	0.794	0.739
	Logistic remove non-outcomes lost to follow-up	0.748	0.649	0.827
	Cox keep lost to follow-up	0.773	0.777	0.781
Hypotension incident event (4385/1877)	Logistic remove lost to follow-up	0.793	0.798	0.831
	Logistic keep lost to follow-up	0.797	0.812	0.811
	Logistic remove non-outcomes lost to follow-up	0.772	0.7	0.873
	Cox keep lost to follow-up	0.798	0.803	0.841
Constipation incident event (7865/3934)	Logistic remove lost to follow-up	0.69	0.684	0.727
	Logistic keep lost to follow-up	0.688	0.708	0.673
	Logistic remove non-outcomes lost to follow-up	0.663	0.569	0.791
	Cox keep lost to follow-up	0.694	0.691	0.738
Diarrhea incident event (9047/4606)	Logistic remove lost to follow-up	0.674	0.659	0.705

## Appendix 3

The simulation equations for the partially synthetic data:

Define $\widehat{X}$ as a ${n}_{1}$ by p matrix where ${n}_{1}$ is the number of patients with complete follow-up and p is the number of predictors. Let $\widehat{{\varvec{y}}}$ be a ${n}_{1}$ by 1 vector such that $\widehat{{y}_{i}\in }$ {0,1}, indicating whether patient i had the outcome during the observed time-at-risk (1 if they did and 0 otherwise).

Define $\stackrel{\sim }{X}$ as a ${n}_{2}$ by p matrix where ${n}_{2}$ is the number of patients with incomplete follow-up and p is the number of predictors. Let $\stackrel{\sim }{{\varvec{y}}}$ be a ${n}_{1}$ by 1 vector such that $\widehat{{y}_{i}\in }$ {0,1}, indicating whether patient i had the outcome during the observed time-at-risk (1 if they did and 0 otherwise).

To simulate complete follow-up, we trained a LASSO logistic regression model using the patients with complete follow-up ($\widehat{X},\widehat{{\varvec{y}}}$) that maps from the predictor space to and value between 0 and 1 indicating the risk of having the outcome$f:\widehat{{\varvec{x}}}\to [\mathrm{0,1}]$. We then applied this to the patients with incomplete follow-up:

Where $f({\stackrel{\sim }{x}}_{i})$ is the predicted risk of the ith patient with incomplete follow-up having the outcome during the time-at-risk. For each patient with incomplete follow-up we simulated their outcome label as:

$$\tilde{y}_{i} = \left\{ {\begin{array}{*{20}l} 1 \hfill & {if\quad f\left( {\tilde{x}_{i} } \right) \ge r_{i} } \hfill \\ 0 \hfill & {else} \hfill \\ \end{array} } \right.,\quad where\quad r_{i} \sim U\left( {0,1} \right)$$

That is, if their predicted risk was greater than a random uniform number between 0 and 1 then they were simulated to have the outcome, otherwise they were simulated to not have the outcome.

In addition, for all patients with incomplete loss to follow-up who were simulated to have the outcome, we simulated the time from index to the outcome by: TAR x ${{r}_{j}, r}_{j} \sim U(\mathrm{0,1})$, where TAR was the full then time-at-risk in days. Effectively, we randomly picked a time during the time-at-risk using a uniform distribution.

We then combined $\widehat{X}$ and $\stackrel{\sim }{X}$ to get the full predictor matrix for all patients and we combined $\widehat{{\varvec{y}}}$ and $\stackrel{\sim }{{\varvec{y}}}$ to get a full vector of labels. This gave us our partial synthetic data.

## Abbreviations

AUROC: Area under the receiver operating characteristic
HER: Electronic health record
IRB: Institutional review board
LASSO: Least absolute shrinkage and selection operator
NLP: Natural language processing
