# Supplementary material for: An empirical analysis of dealing with patients who are lost to follow-up when developing prognostic models using a cohort design
Source: BMC Med Inform Decis Mak. 2021 Feb 6;21:43. doi: 10.1186/s12911-021-01408-x (PMC7866757; doi:10.1186/s12911-021-01408-x)
Supplement: Supplementary file 1 — Additional file 1. The logical definitions for the 21 outcomes used in the empirical real-world data studies. [file 12911_2021_1408_MOESM1_ESM.docx]

Outcome definitions

## Gastrointestinal hemorrhage

Condition occurrence of Gastrointestinal hemorrhage

- for the first time in the person's history
- condition type is any of Inpatient detail - primary, Inpatient header - primary, Primary Condition, Inpatient detail - 1st position, Inpatient header - 1st position
- visit occurrence is any of: Emergency Room Visit, Inpatient Visit

## Acute myocardial infarction

Condition occurrence of Acute MI

- for the first time in the person's history
- condition type is any of Inpatient detail - primary, Inpatient header - primary, Primary Condition, Inpatient detail - 1st position, Inpatient header - 1st position
- visit occurrence is any of: Emergency Room Visit, Inpatient Visit

## Stroke

a condition occurrence of Ischemic stroke

- for the first time in the person's history
- visit occurrence is any of: Inpatient Visit

## Suicide and suicidal ideation

a condition occurrence of Suicide and suicidal ideation

- for the first time in the person's history

**or** an observation of Suicide and suicidal ideation

- for the first time in the person's history

## Insomnia

a condition occurrence of Insomnia

- for the first time in the person's history

## Diarrhea

a condition occurrence of Diarrhea

- for the first time in the person's history

## Nausea

a condition occurrence of Nausea

- for the first time in the person's history

## Hypothyroidism

First valid condition occurrence of Hypothyroidism

inclusion criteria:

- at least 1 occurrences of a condition occurrence of Hypothyroidism

where event starts between 1 days After and 90 days After index start date

## Constipation

a condition occurrence of Constipation

- for the first time in the person's history

## Seizure

a condition occurrence of Seizure and seizure disorder

- for the first time in the person's history
- visit occurrence is any of: Emergency Room Visit, Inpatient Visit

## Delirium

a condition occurrence of Delirium

- for the first time in the person's history
- visit occurrence is any of: Emergency Room Visit, Inpatient Visit

## Alopecia

a condition occurrence of Alopecia

- for the first time in the person's history

## Tinnitus

a condition occurrence of Tinnitus

- for the first time in the person's history

## Vertigo

a condition occurrence of Vertigo

- for the first time in the person's history

## Hyponatremia

a condition occurrence of Hyponatremia

- for the first time in the person's history

OR a measurement of Serum sodium

- for the first time in the person's history
- with value as number between 1 and 136 (inclusive)
- unit is any of: millimole per liter

## Decreased libido

a condition occurrence of Decreased libido

- for the first time in the person's history

## Fracture

a condition occurrence of Fracture

- for the first time in the person's history

## Hypotension

a condition occurrence of Hypotension

- for the first time in the person's history

## Acute liver injury

a condition occurrence of acute liver injury

- for the first time in the person's history
- visit occurrence is any of: Emergency Room Visit, Inpatient Visit
- exactly 0 occurrences of a condition occurrence of acute liver injury exclusions (Disorder of gallbladder, pancreas, biliary tract, Cholecystitis, Gallstone, Chronic hepatitis, Viral hepatitis, liver cancer) between 365 days Before and 60 days After index start date

## Ventricular arrhythmia and sudden cardiac death

a condition occurrence of Ventricular arrhythmia and sudden cardiac death

- for the first time in the person's history
- condition type is any of Inpatient detail - primary, Inpatient header - primary, Primary Condition, Carrier claim detail - 1st position, Carrier claim header - 1st position, Inpatient detail - 1st position, Inpatient header - 1st position, Outpatient detail - 1st position, Outpatient header - 1st position
- visit occurrence is any of: Emergency Room Visit, Inpatient Visit
